# Remarks on the History and Treatment of Delirium Tremens

**Published:** 1832-02

**Authors:** John Ware

**Affiliations:** Fellow of the Massachusetts Medical Society.


### DELIRIUM TREMENS.

***Remarks on the History and Treatment of Delirium Tremens.*** By John Ware, m.d., Fellow of the Massachusetts Medical Society.*

The disease usually denominated Delirium tremens, if not of late origin, has been but lately at least recognised and described as a distinct disease; and the numerous papers which appear relating to it in the periodical Journals shew it to be the general feeling that the subject of its history and treatment has not yet been exhausted.

* From the Transactions of the Massachusetts Medical Society.

The value of any publication on a practical subject can only depend on the extent of the experience of the author, and on the degree of care with which his observations have been made. It is proper, therefore, to state that the cases upon which the following remarks are founded have been observed during the last fourteen years in private practice, and in the practice of the Almshouse of this city: they have amounted in the whole to between ninety and one hundred; of which seventy-seven occurred in private practice, and about twenty in the Almshouse. In these cases, the peculiar symptoms of delirium tremens were clearly, if not always fully developed. I have, in addition to these, in common with all other practitioners, witnessed a large number of cases of disease in drunkards, which, in some stage of their progress, indicated a greater or less tendency to pass into delirium tremens.

It is my object to state the results, with regard to the history and treatment of this disease, which have been derived from these cases, without any particular examination of the works or opinions of those who have already written.

It seems to be generally admitted among physicians, that cases of this disease are by no means always alike; that, though they may agree very well in those peculiar symptoms which are characteristic of it, yet they differ widely from one another in some subordinate, or, perhaps I ought rather to say, less obvious particulars. It has not, however, seemed to be the common opinion that the consideration of this difference should lead to any corresponding difference in the mode of treatment: indeed, it has been the general belief that the most prominent symptoms were those to which the principal regard was to be had in the administration of remedies; and, consequently, physicians have usually attempted the removal of the delirium and watchfulness, leaving such other affections as might accompany these to the chance of relief which the efforts of nature might afford, or, at least, deferring any attention to them till what they regarded as the more important part of the case had been subdued.

The result of such observations as I have been able to make has led to a conclusion somewhat different from this; to a belief, indeed, that the most peculiar and prominent symptoms in cases of this sort are not those to the removal or alleviation of which our efforts are to be chiefly directed. On the other hand, I believe we are mainly to be governed by a reference to such circumstances in the situation of the patient, in the character of his disease, or in the state of his constitution, as would require attention were he not affected with delirium tremens. Still it is not intended to deny that the presence of this affection may, and should, frequently modify the principles on which we proceed in the treatment of such other difficulty as the patient may labour under.

There is hardly any state or degree of disease in drunkards in which an attack of this disease may not be looked upon as a possible, or even probable occurrence. Generally speaking, the more severe the original affection, the more likely is this secondary one to make its appearance: but this is not always true: it supervenes on a very slight indisposition in one individual, whilst another will pass through an attack of great severity without exhibiting any indication of its approach; neither does the degree of indulgence in the use of ardent spirits afford any rule for measuring the probability of its occurrence. It often happens that the confirmed sot will escape its visitation for years, and perhaps for life; whilst a young man, who has but just begun the habit of indulgence may have an attack on the slightest indisposition.

Much of this difference depends, no doubt, on the constitution of different individuals: some are much more susceptible to the immediate or intoxicating effects of alcoholic stimulants than others; and so, too, some seem to be more liable to suffer from their indirect operation, or that which produces disease. Still it does not appear that those who are in the first instance most sensibly affected by the stimulus of ardent spirits are more likely to have disease produced as a consequence of their use than others.

This difference depends partly also on the manner in which the habit of intemperance has been formed: those who become intemperate early in life, and give themselves up almost at once to unlimited indulgence, are commonly broken down within a few years; their constitutions fail them in early manhood, and, among other bad consequences, they are peculiarly liable to attacks of delirium tremens. Those, on the contrary, who have formed the habit gradually, who have used ardent spirits moderately in the first instance, and have come slowly to their excessive use, do not suffer nearly as much in health and constitution, do not sink so easily under the same degree of excess, and are less liable to be affected by the disease of which we are speaking.

It is a common belief that delirium tremens is immediately occasioned by abstinence from ardent spirits, whether this abstinence be forced or voluntary. It is not intended to deny that abstinence may sometimes produce this effect; yet

I feel very certain that, in a large proportion of cases, it has nothing to do with it. The symptoms of this affection frequently ensue shortly after a course of excessive indulgence. In this case it is not that the discontinuance of the indulgence occasions the disease, but that the access of the disease creates a distaste for liquor, and is the occasion of the discontinuance of its use. The disease occurs also in individuals whose habit of drinking has never been suspended at all, but has continued up to the very commencement of the delirium. It happens, also, that a few glasses of spirit will be sufficient to induce a temporary attack of it in an individual in whom it may be said to have existed in a chronic and intermitting form, or in whom, at least, there is so strong a predisposition to a delirium of this character, that very slight causes are sufficient to excite it.

In persons sick with acute diseases, who suffer in the course of them from delirium tremens, it makes its attack, not commonly on the access of the original complaint, but after it has continued some days, and frequently when it has apparently begun to subside. Hence its commencement will be, in most cases, at a considerable period after the use of liquor has been suspended; since, in acute diseases, the propensity for it is either lost, or so much diminished as to lead the patient to relinquish it of his own accord, or to make him at least ready to do so when directed by a physician. It is common, in such a case, for the occurrence of the delirium to be attributed to the suspension of the accustomed stimulus, and from this circumstance, perhaps, the general belief has arisen.

Although delirium tremens occurs in various states of the constitution, and in various diseases, and is to be looked upon as a possible event in almost all cases of indisposition among drunkards, yet there is a remarkable similarity in the phenomena presented by the affection, and in the course of symptoms through which it passes, whatever may have been the original state of constitution or disease from which it has proceeded. Its approach is often indicated by the existence of certain symptoms, from the very commencement of indisposition. It is particularly likely to take place in those who have suffered from irritability of the stomach and frequent vomiting: indeed, it often makes its appearance after having been preceded by no other symptom of disease, and comes on as soon as the vomiting ceases. There is commonly, also, in the beginning of those cases in which delirium finally ensues, a tremor of the hands and limbs, and more frequently of the tongue; a tremulousness of voice producing some indistinctness of articulation; a general anxiety, a hurried manner of moving and speaking, imperfect and disturbed sleep, and startings and twitchings of the limbs. These signs are by no means infallible; they are sometimes observed where delirium does not follow: but, where they exist from the very first, are not diminished by the treatment adopted, and do not leave the patient with the other symptoms of his complaint, an attack of delirium tremens may be reasonably expected.

But, on the other hand, it frequently happens that the attack is not indicated by any such symptoms in the early history of the case. The patient appears to be getting on perfectly well, and the original disease to be subsiding in a satisfactory manner, when suddenly it becomes manifest that an attack of delirium tremens is threatened. In either case, however, whether there have been any premonitory symptoms or not, the disease follows very much the same course. The patient first complains that he has not slept well, that he has been disturbed all night by unpleasant dreams, that he has been hard at work, but that matters have not gone right; that his concerns have troubled and perplexed him. During the next day, perhaps, he is tolerably comfortable, has some appetite, moves about his house or place of business; yet he is uneasy and restless, and exhibits those appearances which have been already described as indicating the approach of the disease. This continues for one or two days; each night being worse than the preceding, whilst in the day there is an increase of the anxiety, restlessness, and trembling of the limbs, tongue, and voice.

The night is then passed with only one or two short naps, from which the patient awakes with some strong impression upon his mind, of the fallacy of which it is difficult or impossible to convince him. His sleep has been filled with dreams of dangers, and perplexities, and annoyances, innumerable and indescribable. From this state he passes into that of complete watchfulness and delirium: the dreams of his sleeping become the fancies of his waking hours, and in his delirium he conceives himself to be engaged in the same occupations, beset by the same difficulties, and surrounded by the same dangers, that he has described as giving a character to his dreams. In fact, it is difficult, in many cases, to point out the precise time at which the mind passes from the dominion of the conceptions which have been engendered in sleep, to that of those which are the offspring purely of the disease.

At whatever period this state of entire watchfulness and delirium begins, we are to date from it the commencement of what may be denominated a paroxysm of delirium tremens.* Yet it will sometimes happen that, on the morning succeeding the night, from the last-continued sleep of which we are to date the commencement of the paroxysm, the patient does not exhibit any unequivocal marks of the delirium by which he is affected. The attendants inform us that he has had but little sleep, and has been very crazy; but we find him sufficiently rational to give an account of his feelings, and fully aware of whatever is going on about him: still his aspect and manner are such as to convey to the mind of one accustomed to the disease the true state of the case, even although there may be no actual exhibition of delirium during the period of the visit.

Most frequently, however, at this time there are occasional wanderings of mind, though not a continued state of delirium. Thus, while sitting by the patient, we perceive his eye become intently fixed upon some remote spot in the room, or without a window, as if it had been suddenly caught by some remarkable object; or he will speak in a loud and quick voice, as if making answer to some one who has addressed him from without, or from behind; or he will start up hastily from his seat or from the bed, and run to another part of the room, or to look beneath the bed, as if in pursuit of something. These impressions are, during the early part of the day, evanescent; but in the latter part the delirium returns, and becomes constant: it increases in violence till about the middle of the night, and then diminishes towards the morning.

On the morning of the second day the delirium is still complete, and is not altered in its character; but the patient is milder and more tractable than during the night: he is as fully possessed of the strange imaginations which have entered into his mind, but he is more easily influenced by his friends, and is more amenable to authority. The second night is generally worse than the first, and there is less abatement of the disease in the ensuing, or third morning, and in the early part of the day; still there is some alleviation of symptoms, like that of the day before. The third day is

* Some objection may probably be thought to lie against this use of the term paroxysm, in which it is made to include a course of symptoms extending through several days. I do not know of any other which would express the intended meaning better. By a paroxysm of delirium tremens, I mean to include that period of a disease of drunkards which is characterised chiefly by delirium of a peculiar kind, and (with rare exceptions) by entire watchfulness, which continues for a certain period, generally not less than sixty or more than seventy-two hours, and terminates either in a critical sleep or in death.

passed much in the same way as the second; but, if the disease is to have a favorable termination, the delirium of the third night is less violent than that of the preceding, and the paroxysm terminates in sleep, sometimes in the course of the evening or first part of the night, but most commonly not until the latter part of the night or in the morning. When the disease is about to terminate unfavorably, the delirium continues undiminished until the fatal event takes place.

This description has been taken from cases which were left to take their own course, uninfluenced by medicine. In all essential points, it will apply to a majority of cases; still there are many variations in the time of day at which the paroxysm begins and terminates, in its length, and in other particulars, which cannot be included under any general account. Thus its duration is sometimes less and sometimes greater than that assigned to it: especially it is apt to be prolonged in those who have had repeated attacks, and in one such case I have known it to extend to nearly six entire days.

During the first part of his sleep, the patient is generally uneasy and restless; his breathing is irregular, and is sometimes almost like that of a person dying. During the first few hours, he often wakes once or twice, perhaps gets up and renews the exercises of his delirious state, or else takes merely a little drink; but, in either case, goes soon to sleep again.

Soon after getting into a sound sleep, the breathing becomes deep, slow, and sonorous: a profuse sweat breaks out, and for a long time the whole body is bathed with it. After six or eight hours, the patient awakes tolerably rational, and sensible of what is going on about him, but generally with some impression left on his mind of the imaginary scenes through which he has passed. He continues, for the next twenty-four, or even forty-eight, hours, to sleep during the greater part of the time: at the end of that period his restoration appears complete, so far as the peculiar symptoms of delirium tremens are concerned; for he may still be the subject of other affections which have preceded the paroxysm, and which remain after it has subsided.

Almost invariably the occurrence of sleep at the close of the paroxysm is indicative of a favorable termination: in some rare cases, however, the patient actually dies after falling asleep, particularly where sleep has been procured by opium: indeed, the only cases which I have seen or known, in which the disease has terminated in this way, have been treated by large doses of opium. In such a case, no peculiar symptoms indicate a different result from that which we usually promise ourselves when the patient falls asleep, till after sleep has taken place: but then, instead of gradually passing from a disturbed into a more tranquil and natural slumber, he becomes first more unquiet and restless, moans, breathes with difficulty, and falls at length into a state of complete coma, from which he never awakes.

The disease terminates fatally in several other ways: sometimes the patient is carried off by the sudden accession of convulsions, and this event is particularly to be looked for in those cases which have begun with them; they also occur very unexpectedly in cases which promise favorably, and which have afforded no ground for anticipating them; sometimes the patient, after continuing the violent exertions of his delirium to the very last moment, without any of the peculiar signs of approaching dissolution, falls back, and expires immediately: sometimes, during the continuance of the delirium, death comes on from the effects of some disease with which it happens to be complicated, and dissolution occurs in the same way that it would from that disease alone.

This is the general course of the phenomena which are exhibited during a paroxysm of delirium tremens: a more particular account of the individual symptoms will be given hereafter. As has been already intimated, they occur under circumstances very various, and in connexion with states of the system and of disease very different from each other: they are accompanied, in these different cases, with different degrees of danger, and require considerable modifications of treatment. Still there is a remarkable similarity in the general course of the paroxysm, and in the conduct and aspect of the patient, between cases the most slight and the most dangerous; between those which arise in a healthy individual, simply from his usual habit of indulgence in intemperance, and those which take place in the course of the most grave and unmanageable diseases: indeed, from seeing the patient only whilst under the influence of the paroxysm, it would be impossible to determine, with any thing like certainty, what his original disease really was; and whether the symptoms which he exhibited were primary, or had arisen secondarily in the course of some other disease.

It is, however, very important, before making up an opinion concerning the probable cause and event, or proper treatment, of any case, to determine the nature of the original affection, to ascertain the precise amount and extent of disease with which we have to contend. I shall proceed, therefore, to describe, as far as my observation furnishes me with materials, the several circumstances, states of the system and of disease, in connexion with which the symptoms of delirium tremens make their appearance.

I. Delirium tremens occurs as the immediate consequence of a particular excess, or of a succession of excesses, in individuals not otherwise disposed to disease. The kind of excess referred to is not simply that habitual use to which all the subjects of this disease have been accustomed, though this is, under certain circumstances, sufficient to its production; but a degree of it, for a short period, somewhat beyond their common habit. It occurs most frequently in such persons as have from any cause been induced or obliged to abstain from the use of ardent spirits for a considerable time, and have again had free access to them: hence it is often seen in sailors after a long voyage, or in those who have been permitted to go on shore from a vessel of war for a few days; but it may also occur in any intemperate person, who, without having previously intermitted the use of spirits, has been tempted to a course of unusual indulgence for several days in succession. Thus it is very common in those who are taken up in a state of intoxication by the civil authority, and committed to almshouses or houses of correction for actual drunkenness. This form of the disease is usually denominated by the vulgar *the horrors;* but the same name is frequently given to the other more severe and aggravated cases.

A considerable number of cases of this description fell under my observation at the Boston Almshouse, when that place was made the receptacle of persons taken up in a state of intoxication. The usual symptoms of delirium manifested themselves in a period varying from a few hours to one or two days from the time of entrance: they were not less severe, not less distinctly marked, than those which occur in the more important cases; but the paroxysm did not uniformly continue for so great a length of time: it sometimes subsided spontaneously in twenty-four hours, though more frequently running out to the full length which has been spoken of as common to the disease generally.

The patient is left in his usual state of health, and, so far as I have ever known, the termination is always favorable. I do not believe that the course of the disease is made shorter, or a fortunate issue more certain, by any mode of treatment whatever: indeed, the employment of many active remedies, particularly of opium, has rather a tendency to aggravate the symptoms than to diminish them.

The occurrence of cases of this class in large numbers in the practice of particular physicians has given occasion to the expression of the opinion that delirium tremens is capable of being almost uniformly treated with success, and that too by very opposite remedies; sometimes by very powerful, sometimes by very simple, modes of treatment. Now, were all the cases of this class treated by opium, they would probably all recover, and if they were not carefully distinguished from cases of a different description, they would confirm, in the mind of the practitioner, the belief that delirium tremens was always readily cured by opium. The same inference might be drawn with regard to any other remedy, which should be made use of in any considerable number of cases: but no inference could be more unfounded; the disease subsides of itself, unaided by art. On this account, statements of the degree of mortality of the disease, as it occurs in public institutions, and of the efficacy of particular remedies in its removal, are to be received with caution.

II. Delirium tremens occurs, secondly, as the consequence of habitual intemperance, without being occasioned by any particular or extraordinary excess, and in this case it approaches more nearly than in any other to the character of an idiopathic disease. It might, indeed, be questioned whether there be sufficient ground for making any distinction between this form of the complaint and the preceding; whether the difference is not merely a difference of severity, in cases essentially the same in nature and character. Probably the state of the brain and the nervous system, upon which the prominent and characteristic symptoms of the paroxysm depend, is essentially the same in all cases: but there is a wide difference in the state of the system at large, and in the symptoms by which it is preceded and followed, and sometimes accompanied; and also in the danger which attends it. This consideration is a satisfactory reason for the arrangement here adopted.

Cases of this kind not only occur without particular acts of excess, but are not unfrequently preceded by an abstinence for several days; the approach of disease sometimes rendering the patient indisposed to his usual indulgence, and sometimes leading him to fear that it may increase the severity and danger of his complaint. The attack does not always take place in precisely the same way, and yet the general course which the symptoms follow is not strikingly different. Sometimes the digestive organs are first affected in a manner very common in the intemperate; sometimes the approach of the disease is like that of a febrile affection; and sometimes the head is primarily affected. More frequently there is a combination of the symptoms arising from these various sources: the patient has chills, followed by heat and sweating; the skin afterwards continues in a moist state, and is usually cool, particularly on the extremities; various sensations are complained of in the head, viz. headach, commonly slight, sometimes very severe, dizziness, and a feeling of confusion and uneasiness when there is no pain, the patient allowing that his head does not feel exactly as in health, although he cannot describe the sensations from which he suffers; the sleep is imperfect, irregular, and unquiet; the tongue is slightly furred, or red and smooth; the appetite usually, but not always, impaired; vomiting frequently occurs; the pulse are usually frequent and wanting in force, sometimes strong, hard, and slow; the eyes are suffused, and the tarsi inflamed: convulsions are occasionally present, and there is commonly a tremulous affection of the muscular system, which exhibits itself in the motions of the eyes, hands, tongue, and diaphragm; there is often, quite early, a little wildness in the expression of the eyes and face; and the mind, though not properly unsettled, is a little removed from its usual state.

None of these symptoms are uniformly present; but the simultaneous existence of a considerable number of them indicates pretty certainly that the patient will finally suffer from delirium tremens. The same symptoms are present, and may lead us to expect a like result in those cases which constitute the third class.

III. But in these they occur not by themselves, but in connexion with other regularly formed and well-marked diseases, or else as the consequence of injuries. In such cases the paroxysm, when it makes its appearance, is, with some exceptions, as distinct in its character and regular in its course, as in those just described; but there are several circumstances connected with the time and mode of its attack, which require particular notice.

The delirium often comes on when the patient is convalescent from the primary disease. In cholera or diarrhœa it may supervene when the vomiting and evacuations from the bowels have ceased, and the weight of the attacks seems to have subsided. In inflammations of the lungs or pleura, after the violence of the symptoms has subsided, after the cough and difficulty of breathing have been relieved, and the pulse have returned to the natural standard, we often perceive, very unexpectedly, the approach of those symptoms which indicate an attack of delirium tremens.

The delirium may also come on when the patient is only apparently convalescent; and it is to be remarked that, in whatever state of the system or of disease an individual is attacked with delirium tremens, any other morbid affection which preexists is absorbed, or at least obscured by it. Thus the apparent convalescence may arise from the obscurity which is thrown over the original symptoms by the approach of those which precede the new complaint. We may erroneously conclude that the patient ceases to suffer, because he ceases to complain; and that the ravages of disease have been checked, because their external indications can be no longer observed. We may learn that this is so, from instances in which the primary disorder is again exhibited after the cessation of the paroxysm of delirium tremens, or in which death takes place during the paroxysm, and dissection shews that it had never ceased to exist.

In other cases there is neither any actual nor apparent improvement before the new attack, the delirium making its appearance at the very height of the original disease: but here also the primary symptoms may be finally absorbed or obscured, so that nothing is afterward apparent sufficient to distinguish it from uncombined delirium tremens, even to a careful observer, who should witness the case in its advanced period only.

There are, however, some cases in which the symptoms of the original disorder continue to be perceptible through the more prominent ones of the paroxysm: the cough, pain, and difficulty of breathing, may be such as to indicate satisfactorily the existence of inflammation of the lungs. In cholera, and more particularly in diarrhœa and dysentery, the continuance of the evacuations after the access of the delirium may bear witness to the continued presence of these diseases, although the pain and exhaustion which they occasion should be obscured by the imaginary sensations and preternatural strength which are the consequences of the new attack.

It is probable that a total change may sometimes take place in the character of the disease under which the system labours, and that the first disease may be actually cured by the attack of the second; an event which is certainly known to happen in other cases. It is not unlikely, also, that a severe acute disease may be attended, from its very first approach, with symptoms of delirium tremens, which might thus veil entirely its character, and conceal its dangers. I am not aware that either of these last-named varieties has been known to occur; but as they are equally probable in themselves as the forms of complication which have been already described, it seems proper to suggest them as cases for the occurrence of which we are to be prepared.

(To be continued.) p. 197.

